# A critical appraisal of transpulmonary and diastolic pressure gradients

**DOI:** 10.14814/phy2.12910

**Published:** 2016-09-01

**Authors:** M. Louis Handoko, Frances S. De Man, Frank P. T. Oosterveer, Harm‐Jan Bogaard, Anton Vonk‐Noordegraaf, Nico Westerhof

**Affiliations:** ^1^ Department of Cardiology VU University Medical Center/Institute for Cardiovascular Research Amsterdam The Netherlands; ^2^ Department of Pulmonary Diseases VU University Medical Center/Institute for Cardiovascular Research Amsterdam The Netherlands; ^3^ Department of Physiology VU University Medical Center/Institute for Cardiovascular Research Amsterdam The Netherlands

**Keywords:** Cardiac output, heart failure, physiology, pulmonary hypertension, pulmonary wedge pressure

## Abstract

Pulmonary hypertension (PH) resulting from left heart failure is an increasingly recognized clinical entity. To distinguish isolated postcapillary PH from combined post‐ and precapillary PH, the use of a diastolic pressure gradient (DPG = diastolic Pulmonary Artery Pressure − Pulmonary Arterial Wedge Pressure, dPAP − PAWP) has been advocated over the transpulmonary pressure gradient (TPG = mean Pulmonary Artery Pressure − PAWP, mPAP − PAWP) since DPG was suggested to be independent of cardiac output (CO) and only slightly related to PAWP, while TPG depends on both. We quantitatively derived and compared the DPG and TPG. Using right heart catheterization data (*n* = 1054), we determined systolic pulmonary artery pressure (sPAP), dPAP and mPAP, PAWP, and CO. From this data, we derived TPG and DPG and tested their dependence on PAWP and CO. We found that dPAP and sPAP are proportional with mPAP over a wide range of PAWP (1–31 mmHg), with dPAP = 0.62mPAP and sPAP = 1.61mPAP. As a consequence, TPG and DPG are equally dependent on PAWP: TPG = mPAP − PAWP, and DPG = 0.62mPAP − PAWP. Furthermore, we showed that both TPG and DPG depend on CO. The absolute increase in DPG with CO is 62% of the TPG increase with CO, but the relative dependence is the same. Both TPG and DPG depend on PAWP and CO. Thus, in principle, there are no major advantages for using DPG to distinguish postcapillary pulmonary hypertension from combined post‐ and precapillary pulmonary hypertension.

## Introduction

Pulmonary hypertension (PH) resulting from left heart failure is an increasingly recognized clinical entity. The presence of PH in left heart failure is described as “isolated post‐capillary PH” when the increase in mean pulmonary artery pressure (mPAP) is solely due to a passive transmission of a high pulmonary arterial wedge pressure (PAWP), with PAWP >15 mmHg (Vachiéry et al. 2014; Galiè et al. 2015). When in left heart failure, the increase in mPAP exceeds the elevation by PAWP, the term “combined post‐ and precapillary PH” (CpcPH) has been proposed; older terms for CpcPH are “out‐of‐proportion PH” or “reactive PH” (Vachiéry et al. 2014; Galiè et al. 2015). CpcPH is defined by an elevated transpulmonary pressure gradient (TPG = mPAP − PAWP >12 mmHg and/or PVR >3WU, with PVR being pulmonary vascular resistance and WU being Wood units) together with PAWP >15 mmHg. Recently, the diastolic pressure gradient has been suggested as a better parameter (DPG = dPAP − PAWP ≥7 mmHg, with dPAP being diastolic PAP) (Vachiéry et al. 2014; Galiè et al. 2015). Stevens (1975) and Enson et al. (1977) originally proposed the use of a DPG, which was recently reintroduced by Naeije et al. (2013). The suggested advantage of DPG over TPG was that: “The transpulmonary pressure gradient (TPG) increases, but the diastolic Ppa/Ppcw gradient (i.e., DPG) is independent of both Ppcw (i.e., PAWP) and SV (stroke volume, and thus cardiac output, CO)”, as mentioned in their Figure 5 (Naeije et al. 2013). Soon, the DPG was tested and applied by several researchers (Al‐Naamani et al. 2015; Haddad and Mielniczuk 2015; Howard et al. 2015).

However, the application of DPG resulted in ambiguous results, as shown in recent discussions in the literature pro/con DPG and TPG (Gerges et al. 2013; Miller et al. 2013; Tedford et al. 2014; Borlaug 2015; Chatterjee and Lewis 2015; Naeije 2015; Tampakakis et al. 2015). In addition, *fundamentally* based comparison with new data has not been carried out. The derivation of DPG by Naeije was based on two equations: (1) dPAP = 0.75PAWP + 3 and (2) mPAP = 1.34dPAP + 0.05SV − 1.3 (Harvey et al. 1971; Naeije et al. 2013). When using this line of reasoning, the magnitude of the coefficient that relates PAWP with dPAP has a major impact on the DPG's dependence or independence on CO and PAWP (see Appendix). Fortunately, as a consequence of the proportionality of sPAP, mPAP, and dPAP for a wide range of PAWP – which we will demonstrate here – the relation between PAWP and dPAP is irrelevant, and the derivation of DPG much more straightforward.

In light of the above, we aimed to:

1. test the basic assumptions on which the DPG and TPG were based, that is, to evaluate the dPAP − PAWP relation and the relation between dPAP and mPAP (Naeije et al. 2013), using original data of over 1000 clinical evaluations of PH patients by means of right heart catheterization performed in our department; and
2. subsequently derive and compare the dependence of DPG and TPG on PAWP and on CO.

We would like to emphasize that no attempt has been made to compare prognostic or diagnostic (dis)advantages of TPG and DPG.

## Methods

### Patients & right heart catheterization

Subjects for clinical evaluation of PH were studied in a stable condition, lying supine, and breathing room air. Out of 1091 hemodynamic evaluations, CO was missing in 37 cases and therefore, analyses were performed on 1054 patients (period 2000–2015). Diagnostic elective right heart catheterization was performed with a fluid‐filled balloon tipped, flow‐directed 7F Swan‐Ganz catheter, via femoral or jugular venous approach. Zero reference pressure level was set with the pressure transducer at midaxillary level. The sPAP, dPAP, mPAP, and PAWP were measured directly at end‐expiration and determined with standard software after visual confirmation of accuracy of the individual tracings. CO was assessed by the Fick method (20% with direct Fick and 8% indirect Fick) or thermodilution (72%). The measurements were carried out under supervision of one researcher (F.P.T.O.) to ensure the quality of the measurements. In case of noninterpretable PAWP, a left ventricular end‐diastolic pressure was obtained and used for PAWP (<5%).

Patients were divided into three groups: (A) subjects suspected of PH but found to have hemodynamics within normal limits: mPAP <25 and PAWP ≤15 mmHg (30%, *n* = 313); (B) mPAP ≥25 and PAWP ≤15 mmHg (56%, *n* = 592); and (C) mPAP ≥25 and PAWP >15 mmHg (14%, *n* = 149).

Due to the retrospective character of this study using data obtained for clinical purposes, the Medical Ethics Review Committee of the VU University Medical Center did not consider this study to fall within the scope of the Medical Research Involving Human Subjects Act. Therefore, no additional approval was acquired.

First, the relation between dPAP and PAWP was determined. Second, relations were derived between sPAP and dPAP with mPAP for different values of PAWP in the range of 1–31 mmHg. These relations were assumed linear and fitted both with an intercept and by forcing the relations through the origin. Since these fits were not significantly different, the linear fits with intercept zero were used in the calculations of TPG and DPG. Based on the relationships between dPAP and mPAP, the following relations between TPG and DPG with PAWP and with CO were derived and tested against the data (see Appendix):

The TPG and DPG dependence on PAWP:

(1a) TPG=mPAP−PAWP

(1b) DPG=dPAP−PAWP=0.62mPAP−PAWP

The TPG and DPG dependence on CO:

(2a) TPG=PVR∗CO

(2b) DPG=0.62PVR∗CO−0.38PAWP

### Statistics

GraphPad Prism for Windows 5.0 (GraphPad Software, San Diego CA) and IBM SPSS for Windows 22 (SPSS Inc., Chicago IL) were used for analyses. All relations were fitted with linear regression. The slope and its standard deviation of the pressure–pressure relations were calculated. A *P* < 0.05 was assumed statistically significant.

## Results

### Patient characteristics

We used a broad spectrum of normal subjects and patients with normal to high pulmonary artery pressures and a large range of PAWP. Patient characteristics according to the subgroups are given in Table 1 (patient characteristics according to WHO classification of PH are given in Table A1). The averaged age and range were 60 (18–87) years, and 66% were female.

**Table 1:** Patient characteristics

	All (*n* = 1054)	Group A: mPAP < 25 mmHg PAWP ≤ 15 mmHg (*n* = 313)	Group B: mPAP ≥ 25 mmHg and PAWP≤15 mmHg (*n* = 592)	Group C: mPAP ≥ 25 mmHg and PAWP > 15 mmHg (*n* = 149)
Gender (% female)	66	67	63	72
Age (years)	60 ± 15	57 ± 15	60 ± 16*	65 ± 13*, #
BMI (kg‐m^2^)	27 ± 7	27 ± 6	26 ± 6	30 ± 8#
BSA (m^2^)	1.9 ± 0.3	1.9 ± 0.3	1.9 ± 0.4	2.0 ± 0.3
Heart rate (/min)	78 ± 15	75 ± 13	81 ± 15*	76 ± 15#
SaO_2_ (%)	94 ± 5	96 ± 3	92 ± 5*	94 ± 5*, #
SvO_2_ (%)	67 ± 10	73 ± 7	64 ± 10*	64 ± 9*
mRAP (mmHg)	7 ± 6	5 ± 6	8 ± 5*	12 ± 6*, #
sPAP (mmHg)	58 ± 26	28 ± 7	72 ± 20*	66 ± 21*, #
dPAP (mmHg)	22 ± 11	11 ± 4	27 ± 10*	26 ± 9*, #
mPAP (mmHg)	36 ± 16	18 ± 4	44 ± 12*	42 ± 12*, #
PAWP (mmHg)	11 ± 5	9 ± 4	9 ± 3	21 ± 4*, #
CI (L/min/m^2^)	3.1 ± 1.2	3.7 ± 1.3	2.9 ± 1.1*	2.8 ± 1.1*
PVR (Wood units)	5.5 ± 4.9	1.4 ± 0.8	7.9 ± 4.8*	4.6 ± 4.2*, #
TPG (mmHg)	25 ± 16	9 ± 4	35 ± 13*	21 ± 12*, #
DPG (mmHg)	11 ± 12	1 ± 4	18 ± 10*	5 ± 9*, #

BMI, body mass index; BSA, body surface area; SaO_2,_ arterial oxygen saturation; SvO_2,_ venous oxygen saturation; mRAP, mean right atrial pressure; sPAP, dPAP, and mPAP, systolic, diastolic, and mean pulmonary arterial pressure; CI, cardiac index; PAWP, pulmonary arterial wedge pressure; PVR, pulmonary vascular resistance; TPG and DPG, transpulmonary and diastolic pressure gradient. Average ± SD, *P < 0.05 versus Group A; #*P* < 0.05 versus Group B.

### Relation between dPAP and PAWP

**Figure 1:**
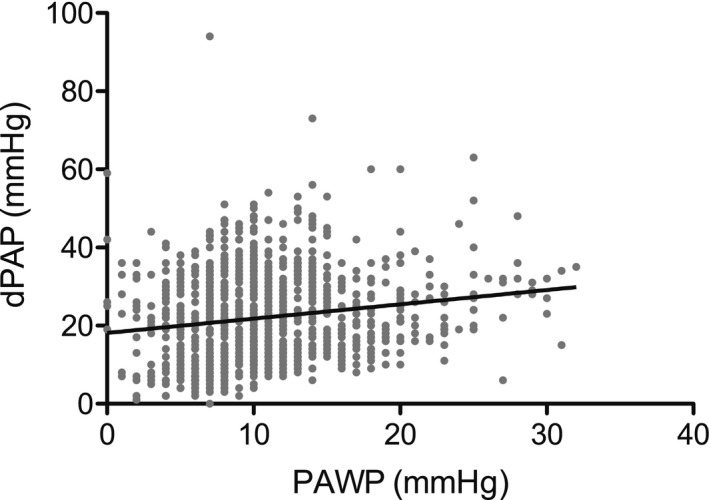
The relation between diastolic pulmonary artery pressure (dPAP) and pulmonary arterial wedge pressure (PAWP): dPAP = 0.37PAWP + 18 mmHg; 95% confidence interval slope: 0.24–0.49, *R* ^2^ = 0.03, *n* = 1054.

The relation between dPAP and PAWP is presented in Figure 1: The relation is weak (*R* ^2^ = 0.03) yet significant, with confidence interval (CI) of the slope being 0.24–0.49. For the three groups A, B, and C, the following relations were found: group A: dPAP = 0.39PAWP + 6.6 (slope 95% CI = 0.29–0.48, *R* ^2^ = 0.17); group B: dPAP = 0.24PAWP + 25.4 (slope 95% CI = 0.01–0.46, *R* ^2^ = 0.01); and group C: dPAP = 0.60PAWP + 13.2 (slope 95% CI = 0.22–0.98, *R* ^2^ = 0.06). Slopes are not statistically different.

### Relations between dPAP and sPAP with mPAP

**Figure 2:**
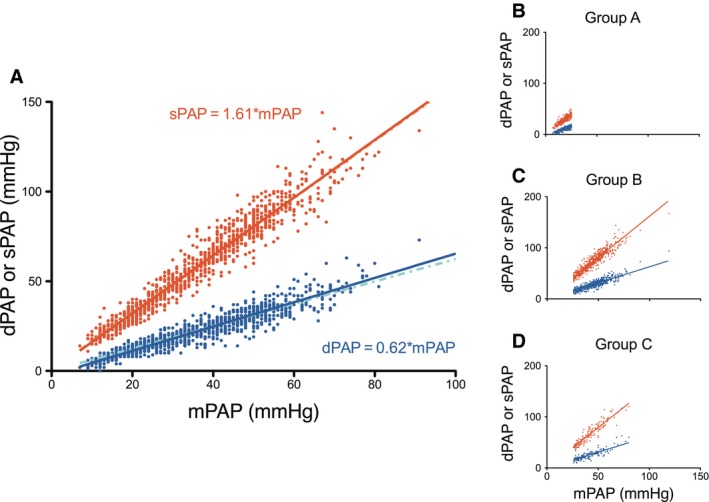
(A) The relation of systolic (sPAP) and dPAP with mean pulmonary arterial pressure (mPAP), *n* = 1054. The relations with intercept (lighter dashed lines) and without intercept (continuous lines) nearly completely overlap, and are visually nearly indistinguishable. (B–D) The relation of sPAP and dPAP with mPAP for the three subgroups of patients are consistent between subgroups. Group A: mPAP < 25 and PAWP ≤ 15 mmHg, *n* = 333; Group B: mPAP ≥ 25 and PAWP ≤ 15 mmHg, *n* = 578; Group C: mPAP ≥ 25 and PAWP > 15 mmHg, *n* = 143. For visual reasons only, the data point of one patient with mPAP > 100 mmHg was not plotted.

**Figure A2:**
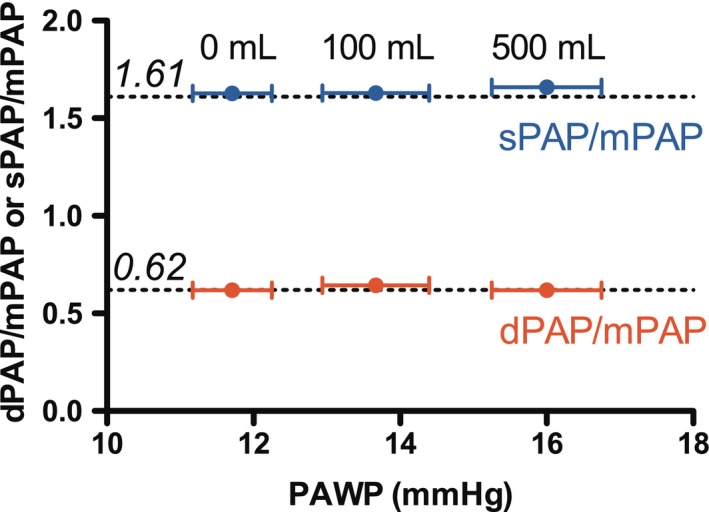
In a subgroup of patients, a fluid‐challenge was performed for diagnostic reasons (*n* = 28). The dPAP/mPAP and sPAP/mPAP ratio remained constant despite increase in PAWP during fluid challenge. The dotted lines mark the overall dPAP/mPAP and sPAP/mPAP ratios that were found for the whole group.

Figure 2A gives the relations between dPAP and sPAP with mPAP for the entire group, with dPAP = 0.62 ± 0.01 mPAP and sPAP = 1.61 ± 0.01mPAP (mean ± SEM). Figure 2B illustrates these relations for the three subgroups A, B, and C. These relations are not different from those of the entire group. In patients with known heart rate (*n* = 963), the ratios sPAP/mPAP = 1.62 ± 0.01 and dPAP/mPAP = 0.61 ± 0.01 (mean ± SEM) were not different from the whole group. In a subset of patients (*n* = 28), a fluid challenge was carried out (500 mL of saline in 15 min), with PAWPs of 11 ± 1 (0 mL) to 14 ± 1 (100 mL) and 16 ± 1 mmHg (500 mL), respectively (see also Fig. A2). The average sPAP/mPAP = 1.64 ± 0.02 and dPAP/mPAP = 0.63 ± 0.01 (mean ± SEM), both not statistically different from 1.61 to 0.62. This data show that the relations also hold *within* patients.

**Figure 3:**
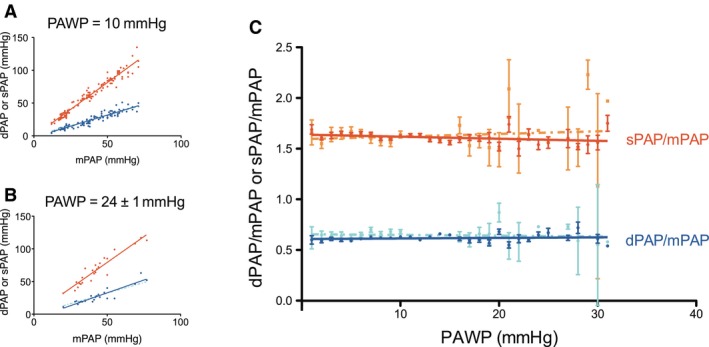
(A–B) The relation of sPAP and dPAP with mPAP in patients with a PAWP of 10 mmHg (*n* = 115) and of 24 ± 1 mmHg (*n* = 24). The relations with intercept (lighter dashed lines) and without intercept (continuous lines) nearly completely overlap. (C) The ratio of sPAP and dPAP relative to mPAP, as function of PAWP; standard errors are indicated.

Figure 3A and B show two examples of the patients with low and high PAWP. The differences between the fits with and without intercept are small. For all PAWP studied (1–31 mmHg), the *R* ^2^ values (average and interquartile range) of the dPAP/mPAP relation was found to be 0.88 (0.77–0.92) and 0.89 (0.79–0.92) with and without intercept, respectively. For the sPAP/mPAP relation, these results were 0.94 (0.90–0.97) and 0.94 (0.91–0.97). The relation between sPAP and mPAP is significantly tighter than the relation between dPAP and mPAP, as can be judged from the *R* ^2^ values (*P* > 0.01). Figure 3C shows the dPAP/mPAP and sPAP/mPAP ratios as a function of PAWP for the entire range of PAWP studied (1–31 mmHg). The ratios does not depend on PAWP, and were not different from the average values reported in Figure 2A, as demonstrated using the following analysis: dPAP/mPAP‐ratio versus PAWP: no intercept slope = 0.0005 ± 0.0008 (*P* = 0.48, not different from horizontal/slope = 0) and with intercept slope = −0.0007 ± 0.001 (*P* = 0.60). There was also no difference between the relations with and without intercept. Therefore, the slopes without intercept were used in the derivation of DPG that will follow.

### How TPG and DPG depend on PAWP

**Figure 4:**
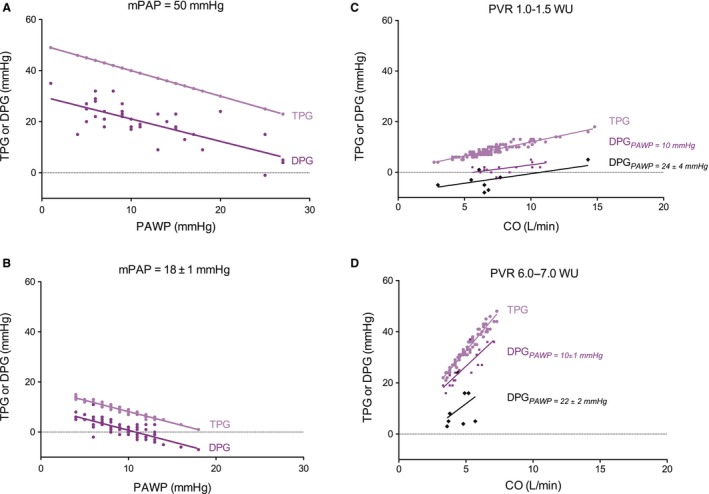
(A–B). Relations between transpulmonary pressure gradient (TPG) and diastolic pressure gradient (DPG) as a function of PAWP, for a low (A, *n* = 77) and high (B, *n* = 38) value of mPAP. All slopes do not differ from the predicted slope‐1. (C–D) Relations between TPG and DPG and cardiac output (CO), for two different values of pulmonary vascular resistance (PVR): (C) Average PVR = 1.2 Wood units (WU). TPG,* n* = 106; DPG and PAWP 10 mmHg, *n* = 16; DPG and PAWP 24 ± 4 mmHg, *n* = 9; (D) Average PVR =6.5WU. TPG,* n* = 67; DPG and PAWP 10 ± 1 mmHg, *n* = 18 DPG and PAWP 22 ± 2 mmHg, *n* = 9. Slopes of TPG are equal to PVR. Slopes of DPG are not different from the predicted 0.62PVR. The intercepts with the vertical axis do not differ from the predicted −0.38PAWP.

Since TPG and DPG depend on mPAP and on PAWP, the effect of PAWP is shown for similar mPAP. In Figure 4A and B, two examples are given for patients with a low and high mPAP. Slopes of the relations do not differ from −1, thus, TPG and DPG equally depend on PAWP. For other choices of mPAP, slopes were never found to differ from −1, showing that both TPG and DPG depend equally on PAWP.

### How TPG and DPG depend on CO

Figure 4C and D illustrates the dependence of TPG and DPG on CO. Since TPG depends on PVR and on CO, the effect of CO is given for a low and high PVR. There is indeed a proportional relation between TPG and CO, the slopes being equal to PVR.

The DPG depends on CO and two other parameters, PVR and PAWP. Therefore, the relations are also given for the same two values of PVR, plus a low and high PAWP (Fig. 4C and D). The slopes are not different from each other, but also not different from 0.62 of the slope between TPG and CO. The intercepts depend on PAWP as −0.38PAWP. Since the magnitude of DPG is 62% of the TPG, the *relative* slope of the relation between DPG and CO equals the relative slope of TPG with CO. Of note, DPG was sometime found to be negative (dPAP < PAWP), especially when PAWP is high and PVR/CO low.

**Figure 5:**
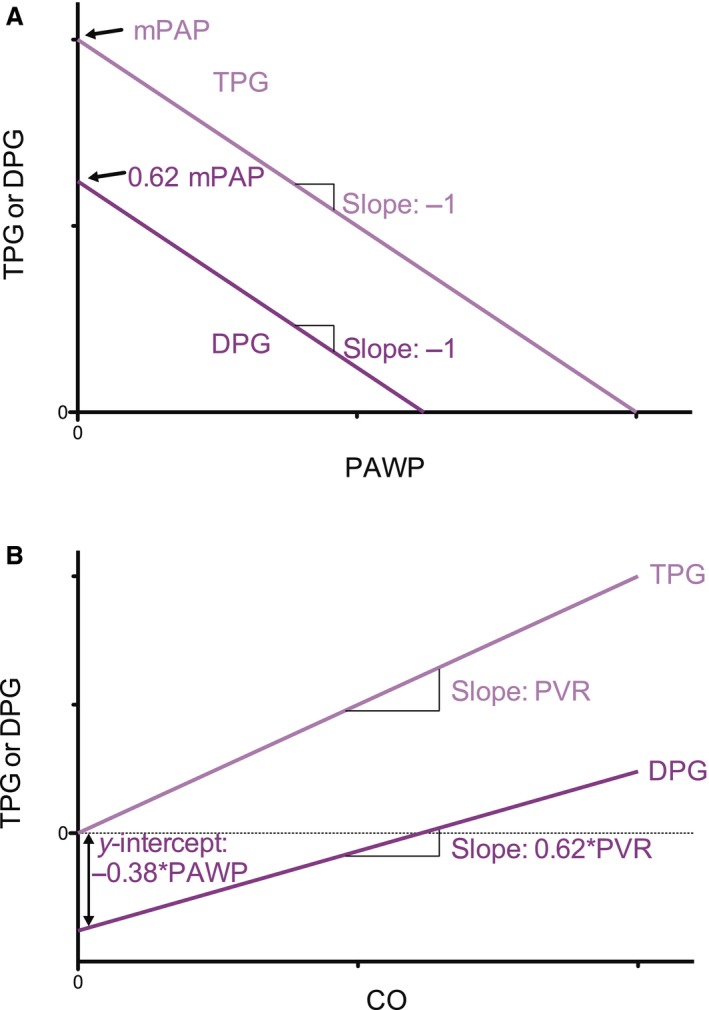
Schematic representation of the dependence of TPG and DPG on (A) PAWP and (B) on CO.

Thus, the data show that TPG and DPG behave as predicted from the (averaged) dPAP/mPAP ratio and as expressed in our equations (1) and (2). The schematic summary of how TPG and DPG depend on PAWP and on CO is presented in Figure 5.

## Discussion

To help in the understanding of TPG and DPG and their differences, we derived the basis on which both depend, namely PAWP and CO. The basis is the PAWP‐*independent* proportionality between dPAP and mPAP (Figs. 2, 3, and A2).

### dPAP and PAWP

Figure 1 shows that dPAP = 0.37PAWP + 18 mmHg; Harvey et al. (1971) reported dPAP = 0.74PAWP + 3 mmHg, and Rahimtoola et al. (1972) found dPAP = 0.72PAWP + 7 mmHg. Averaged data by Gerges et al. (2013) and Miller et al. (2013) are −0.45PAWP + 36.6 and 0.88PAWP + 2.4, respectively. The correlation between dPAP and PAWP that we and others found are therefore inconsistent (ranging from −0.45 to 0.88). These differences are, however, in the context of TPG and DPG inconsequential since the proportionality dPAP = 0.62mPAP is the only basic, and experimentally verified, relation upon which their dependence on PAWP and CO is based (see Appendix).

### Proportionality of pressures

The proportionality between dPAP and mPAP (0.62) is close to the factor 0.6 reported by Miller et al. (2013). The dPAP/mPAP proportionality by Syyed (0.71) and Tampakakis (0.75) differ somewhat from our present results (Syyed et al. 2008; Tampakakis et al. 2015). A value of 0.71 does not qualitatively affect the TPG dependence on PVR and CO. The dependence of DPG on PAWP and CO would change to: DPG = 0.71mPAP − PAWP and DPG = 0.71CO*PVR − 0.29PAWP. In words, the DPG dependence on PAWP remains unaffected, and its dependence on PVR would increase from 0.62 to 0.71.

The proportionality constant between sPAP and mPAP is close to those reported by others (Chemla et al. 2004, 2015; Syyed et al. 2008; Miller et al. 2013). Chemla et al. (2004, 2015) showed that the sPAP/mPAP relation is strong, independent of PAWP, and remaining similar in CTEPH. In his recent literature overview, Chemla et al. (2015) reported the averaged value of sPAP/mPAP = 1.57. Tampakakis et al. (2015) reported about 1.5. The sPAP/mPAP relation does not determine the dependence of TPG and DPG on PAWP and CO.

### DPG and TPG are equally dependent on PAWP

We have shown that TPG and DPG can be derived from the PAWP‐independent relation between dPAP and mPAP. In papers proposing the dependence of TPG and the (relative) independence DPG on PAWP and CO, several relations between dPAP and PAWP were used. It was shown that using Harvey's relation dPAP = 0.74PAWP + 3 (Harvey et al. 1971; Naeije et al. 2013) resulted in (strong) dependence of DPG on PAWP. However, using the relation dPAP = 1.0*PAWP + 3 predicted independence of DPG on PAWP (Naeije et al. 2013; Tedford et al. 2014; Naeije 2015). Thus, a strong effect of the dPAP − PAWP relation on TPG and DPG is suggested. However, we show here that the relation between dPAP and PAWP is not a factor in the derivation of TPG and DPG (see Appendix).

### DPG and TPG both depend on CO

The TPG depends on CO proportional to PVR. The proportionality dPAP = 0.62mPAP, implies that DPG depends on CO (Figs. 4 and 5) with proportionality factor 0.62PVR, with the negative intercept for DPG equaling –0.38PAWP. Since TPG is in magnitude approximately 62% of TPG and the increase of DPG is about 62% of the increase of TPG on CO, their relative dependence on CO is the same.

### Explanation of a negative DPG

We found that, on average, negative DPG (dPAP − PAWP < 0) for PAWP > 29 mmHg (Fig. 4), which was also predicted by our equation (2b). Rahimtoola et al. (1972), Gerges et al. (2013), and Miller et al. (2013) found dPAP < PAWP for PAWP values larger than 25, 25, and 20 mmHg, respectively. Even though a mPAP should always be higher than PAWP, a lower dPAP than PAWP is not impossible, since dPAP is a pressure *at one instant*, which may be momentarily lower than the *mean* PAWP, especially when PAWP is high and CO/PVR low.

### Further comparison between TPG and DPG

The fact that TPG uses mPAP, while DPG uses dPAP implies that DPG is more subject to noise and small measurement errors: mean pressures are less subject to catheter artifact than diastolic pressures. It may be seen in Figure 4 that the DPG‐data scatter more than the TPG‐data. Also, since DPG ≥7 mmHg and TPG >12 mmHg are assumed as CpcPH criterion, it implies that a 1 mmHg error in both is, percentagewise, much larger in DPG than TPG (Nichols and O'Rourke 2005).

Since both TPG and DPG depend on CO, we would favor the use of PVR >3WU together with PAWP >15 mmHg as criterion to distinguish CpcPH from isolated postcapillary PH (Miller et al. 2013; Galiè et al. 2015).

### Limitations

The data was analyzed retrospectively, which makes it more prone for bias. The individual recording from the right heart catheterization have major implications for patient management. For this reason, at our institute, a nurse practitioner (F.T.P.O) was present in >90% of the cases to ensure consistent, accurate, and high‐quality recordings (correct zeroing, wedging, etc.), despite differences in techniques and operators over time. A consequence of our retrospective analysis is determination of CO by different techniques. However, our conclusion that TPG and DPG both depend on CO and PAWP is derived from the observation that the proportionality of dPAP/mPAP was preserved over a very wide range of PAWP, and thus does not directly depend of CO nor how it was measured.

The distribution of patients with precapillary PH, isolated postcapillary PH, and CpcPH is somewhat different from previously reported, depending on which criteria was used (relative high number of CpcPH‐patient based on TPG, lower and more comparable to other studies when DPG is used). This may be a simple reflection of referral pattern, as patients at the VUmc that undergo a right heart catheterization are primary analyzed for suspicion of PH WHO‐group 1, which could result in a relative low number of patients with isolated postcapillary PH. We would like to stress out again that the primary goal of this study is to investigate the principles from which TPG and DPG are derived, rather than a head‐to‐head comparison of DPG versus TPG to distinguish isolated postcapillary PH from CpcPH.

The derivations of TPG and DPG (see Appendix) are based on proportional relations between dPAP and mPAP, that is, neglecting small and statistically insignificant intercepts. Furthermore, the theoretical relations (eq. 1 and 2) are also empirically confirmed by the data presented in Figure 4, suggesting that not taking the intercepts into account, introduces negligible errors.

We have analyzed the relations in terms of CO rather than stroke volume. We made this choice because this is the most straightforward approach. We demonstrated that the pressure–pressure relations in the total group and the subgroup with known heart rates are similar. This was previously also shown by Kind et al. (2011).

## Conclusion

Our finding that proportionality of dPAP and mPAP is maintained for PAWP between 1 and 31 mmHg implies that both TPG and DPG depend equally on PAWP, and that both depend on CO, and in terms of percentage even similarly.

In principle, there are no major advantages to use DPG over TPG to distinguish isolated postcapillary PH from CpcPH. The former criterion TPG > 12 mmHg and other criteria (e.g., PVR >3WU as mentioned in the recent guidelines) for diagnosis and prognosis should be reassessed in a large population.

## Conflict of Interest

None declared.

## Figures and Tables

**Figure A1:**
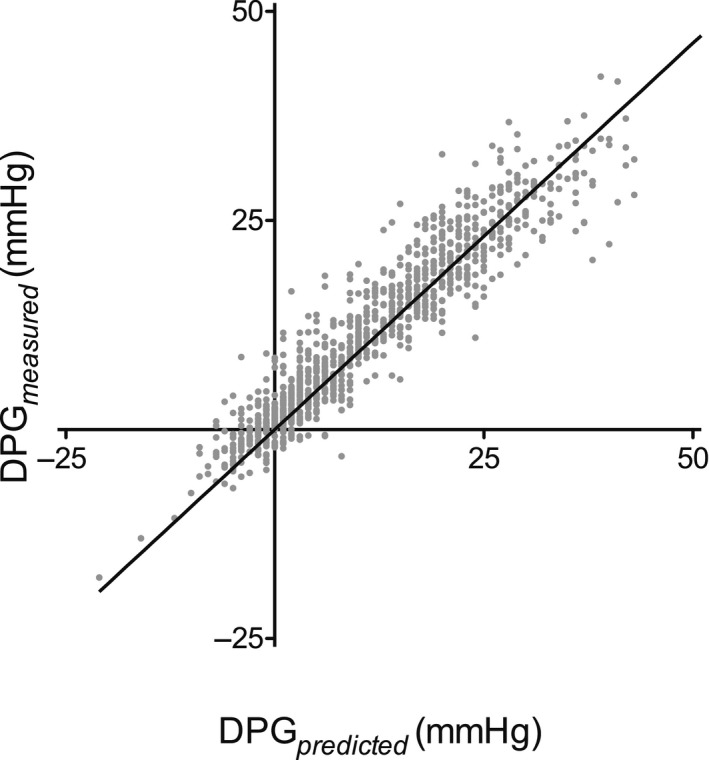
The relation between the predicted DPG (DPG_*predicted*_ = 0.62 PVR*CO − 0.38 PAWP) and the actual measured DPG (*R* ^2^ = 0.89, *P* < 0.001). For clarity, three data points were left out because they were out of range (but included in the statistical analysis).

## Additional calculations:

### Previous derivation DPG

The earlier derivation of DPG was based on two major assumptions (Harvey et al. 1971; Naeije et al. 2013).

dPAP = 0:75PAWP + 3   (Harvey et al. 1971  : 0:743; figure 4 of Naeije 2015 : 0:75)      (a)

mPAP = −1:33 + 1:34dPAP + 0:05SV;   with SV  : Stroke Volume             (b)

Inserting the equation (a) into equation (b) gives:

mPAP = 2.69 + 1.005PAWP + 0.05SV.

The results of TPG and DPG are:

TPG = mPAP – PAWP = 2.69 + 0.05SV + 0.005PAWP

or in approximation: TPG ≈ 2.7 + 0.05SV; and

DPG = dPAP – PAWP = 0.75PAWP + 3 – PAWP = −0.25PAWP + 3.

We see that DPG *depends* on PAWP but TPG *does not*. A result contrary to what has been suggested in Naeije et al. (2013).

However, if it is assumed that dPAP = 1.0PAWP + 3 (figure 5 of Naeije et al. (2013), instead of 0.75PAWP + 3), the results for TPG and DPG are:

TPG = 0.34PAWP + 2.69; and

DPG = 3.

With this assumption, DPG does not depend on PAWP but now TPG does (see figure 5B of Naeije et al. (2013)).

In summary, the assumed relation between dPAP and PAWP determines the dependence of TPG and DPG on PAWP. However, both the relations between dPAP and PAWP are not confirmed by us (our Fig. 1) or others, and this relation is extremely poor and cannot be used as basis for predictions.

The dependence of TPG and DPG on PAWP and CO can be derived from the proportionality of dPAP/mPAP relations that are independent of PAWP, and this derivation does *not* require an assumed relation between dPAP and PAWP (see below).

### How TPG and DPG depend on PAWP

By definition:

(1a) TPG=mPAP−PAWP

and

DPG = dPAP − PAWP.

Thus, with dPAP = 0.62mPAP,

(1b) DPG=dPAP−PAWP=0.62mPAP−PAWP

**Table A1:** Patient characteristics by WHO classification

	All (*n* = 1054)	Control (*n* = 316)	WHO‐group 1 (*n* = 376)	WHO‐group 2 (*n* = 143)	WHO‐group 3 (*n* = 78)	WHO‐group 4 (*n* = 115)	WHO‐group 5 (*n* = 26)
Gender (% female)	66	65	68	76	41	63	65
Age (years)	60 ± 15	58 ± 15	57 ± 17	66 ± 12	65 ± 12	64 ± 14	59 ± 11
BMI (kg‐m^2^)	27 ± 7	27 ± 7	26 ± 6	30 ± 8	26 ± 6	25 ± 7	24 ± 4
BSA (m^2^)	1.9 ± 0.3	1.9 ± 0.3	1.8 ± 0.3	2.0 ± 0.3	1.9 ± 0.2	1.9 ± 0.5	1.7 ± 0.2
Heart rate (/min)	78 ± 15	75 ± 13	81 ± 15	75 ± 15	84 ± 16	77 ± 14	87 ± 17
SaO_2_ (%)	94 ± 5	96 ± 3	93 ± 4	94 ± 4	89 ± 7	93 ± 4	90 ± 9
SvO_2_ (%)	67 ± 10	72 ± 7	64 ± 10	66 ± 10	62 ± 10	62 ± 10	67 ± 9
mRAP (mmHg)	7 ± 6	5 ± 6	8 ± 6	11 ± 6	6 ± 4	9 ± 5	6 ± 4
sPAP (mmHg)	58 ± 26	30 ± 9	74 ± 21	62 ± 21	63 ± 19	73 ± 21	67 ± 18
dPAP (mmHg)	22 ± 11	11 ± 4	29 ± 11	23 ± 8	25 ± 9	27 ± 9	26 ± 10
mPAP (mmHg)	36 ± 16	19 ± 5	46 ± 13	38 ± 12	40 ± 12	44 ± 12	41 ± 11
PAWP (mmHg)	11 ± 5	10 ± 5	9 ± 4	18 ± 6	10 ± 4	10 ± 3	9 ± 4
CI (L/min/m^2^)	3.1 ± 1.2	3.6 ± 1.3	2.8 ± 1.1	3.0 ± 1.2	3.0 ± 0.9	2.5 ± 1.0	4.1 ± 1.3
PVR (Wood units)	5.5 ± 4.9	1.5 ± 0.9	8.5 ± 5.1	3.9 ± 3.0	6.2 ± 4.3	8.3 ± 4.9	5.2 ± 2.8
TPG (mmHg)	25 ± 16	9 ± 5	36 ± 14	20 ± 10	30 ± 12	34 ± 12	32 ± 11
DPG (mmHg)	11 ± 12	1 ± 4	19 ± 11	5 ± 8	15 ± 9	17 ± 8	16 ± 9

BMI, body mass index; BSA, body surface area; SaO_2,_ arterial oxygen saturation; SvO_2,_ venous oxygen saturation; mRAP. mean right atrial pressure; sPAP, dPAP and mPAP, systolic, diastolic and mean pulmonary arterial pressure; CI, cardiac index; PVR, pulmonary vascular resistance; TPG and DPG, transpulmonary and diastolic pressure gradient. Average ± SD.

Figure A1 shows this theoretical prediction with the measured DPG.

These relations show that both TPG and DPG depend equally on PAWP.

### How TPG and DPG depend on cardiac output

Because mPAP – PAWP = PVR·CO, it follows that

(2a) TPG=PVR·CO

With DPG = dPAP − PAWP and dPAP = 0.62mPAP,

DPG = 0.62mPAP – PAWP = 0.62mPAP − (0.62PAWP − 0.38PAWP)

Thus,

(2b) DPG=0.62PVR·CO−0.38PAWP

These relations show that TPG and DPG depend on CO.

Since DPG ≈ 0.62TPG, the relative effect of CO on DPG is the same as the relative effect of CO on TPG.

Remark:

Syyed et al. (2008) reported that dPAP ≈ 0.71MPAP; this does not result in a different sensitivity of TPG and DPG on PAWP. Then, the sensitivity of TPG on CO would change to: DPG = 0.71PVR·CO − 0.29PAWP.
